# A rare clinical case of systemic AA amyloidosis with cardiac involvement complicating ankylosing spondylitis: a case report

**DOI:** 10.1186/s43044-024-00471-9

**Published:** 2024-03-28

**Authors:** Leïla Barakat, Khadija Echchilali, Mina Moudatir, Hassan El Kabli, Yassine Ettagmouti, Mériem Haboub, Salim Arous, Mohamed Ghali Benouna, Abdenasser Drighil, Rachida Habbal, Meryame Azim, Asmae Mazti, Meriem Regragui, Nissrine Bennani Guebessi, Mehdi Karkouri

**Affiliations:** 1 Department of Internal Medicine, CHU Ibn Rochd, Casablanca, Morocco; 2 Cardiology Department, Ibn Rochd University Hospital, Casablanca, Morocco; 3 Laboratory of Pathological Anatomy, CHU Ibn Rochd, Casablanca, Morocco

**Keywords:** Secondary amyloidosis, Ankylosing spondylitis, Cardiac amyloidosis, Amyloid goiters, Case report

## Abstract

**Background:**

Ankylosing spondylitis (AS) is a type of chronic inflammation that is most prevalent in young adults and is characterized by an inflammatory enthesiopathy that gradually develops toward ossification and ankylosis. If inflammation is left unchecked, it can potentially lead to complications such as secondary amyloidosis, also known as AA amyloidosis, involving the deposition of amyloid serum A protein. Our case presents with a thyroid localization of AA amyloidosis which is secondary to this AS. Such a case has been described in only four cases in the literature. Cardiac localization of AA amyloidosis has been exceptionally described in the literature.

**Case presentation:**

We report the case of a young patient with severe AS complicated by secondary amyloidosis with thyroid, cardiac, and probably renal localization. He was treated with anti-TNF therapy, and his condition improved significantly.

**Conclusions:**

Our case presents several localizations of AA amyloidosis secondary to this AS. Although cardiac involvement is rare in secondary AA amyloidosis, it should always be screened for, even in a cardiacly asymptomatic patient.

## Background

Secondary amyloidosis (AA) is an important complication of chronic inflammatory diseases. Rheumatologic diseases such as rheumatoid arthritis (RA) and ankylosing spondylitis (AS) are known to be associated with the development of AA amyloidosis. Although AS affects the spine and sacroiliac joints as well as peripheral joints, other extra-articular manifestations are possible and include acute anterior uveitis, aortic insufficiency, apical pulmonary fibrosis, and systemic amyloidosis. AA is a systemic disease characterized by amyloid deposition in many organs. The kidney is the most affected organ, damage to the thyroid gland is possible, but cardiac involvement remains rare during secondary amyloidosis. The present case illustrates a unique presentation of multisystem secondary amyloidosis.

## Case presentation

**Fig. 1:**
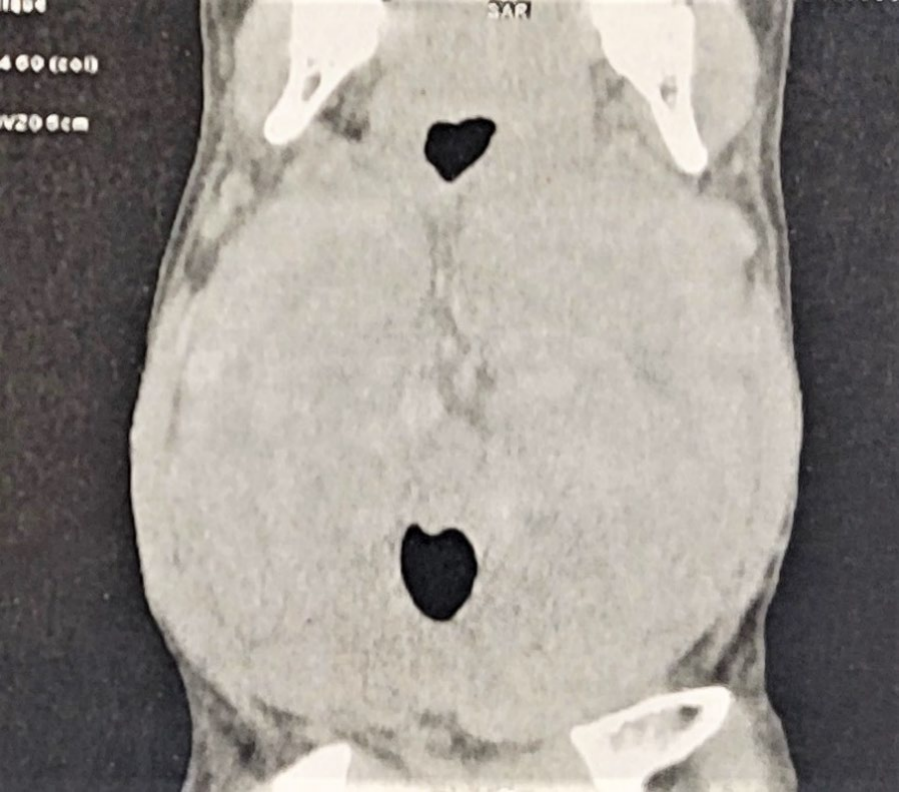
Frontal section of a voluminous goiter compressing the laryngeal canal, bumpy contours with heterogeneous density

**Fig. 2:**
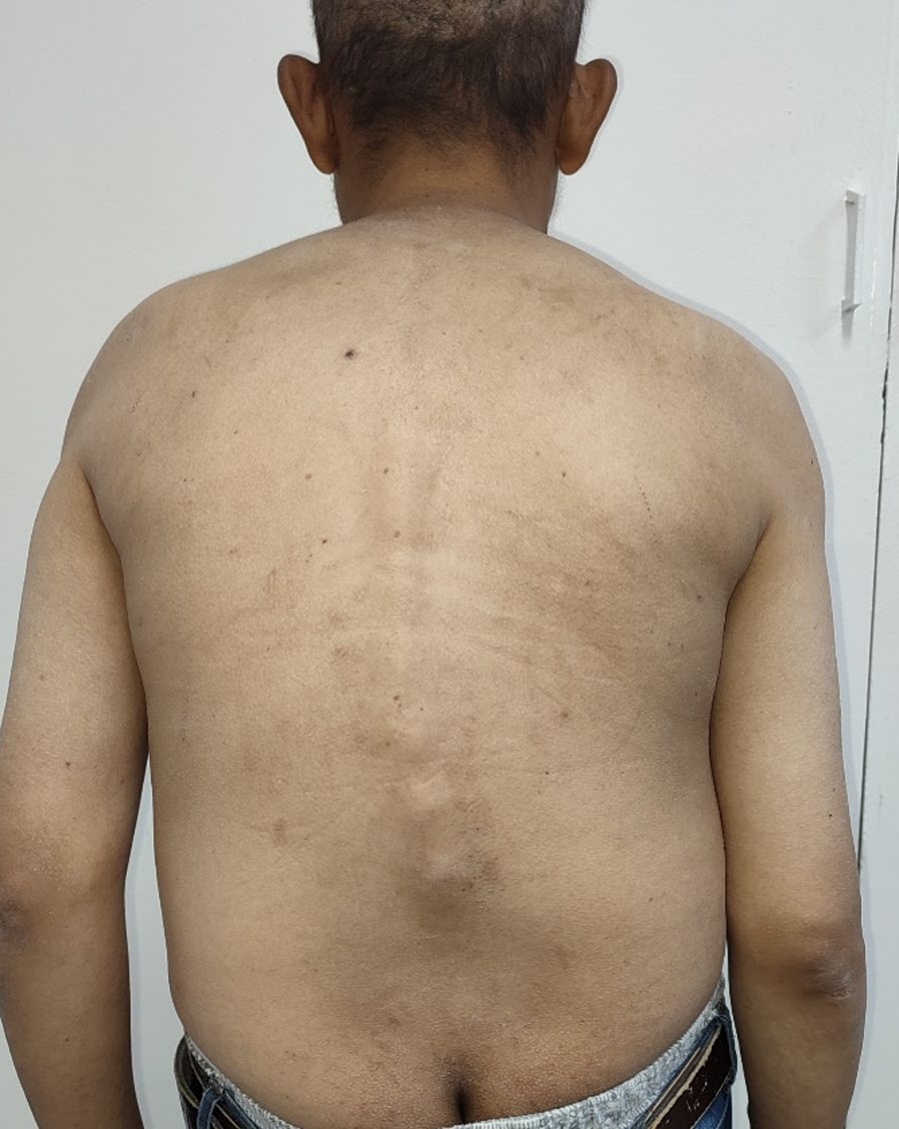
Kypho-scoliosis with spinal stiffness

A 33-year-old male patient, on dialysis since the age of 26 for kidney damage of undocumented origin, referred to an internal medicine consultation for the etiological assessment of a large amyloid goiter (Fig. 1) evolving for 3 years with a normal thyroid balance initially. During the interrogation, the patient reported since the age of 16 years inflammatory low back pain associated with buttock pain, heel pain, and enthesopathy, which prompted the patient to self-medicate with nonsteroidal anti-inflammatory drugs if necessary. On clinical examination, the patient presented with kypho-scoliosis, stiffness of the cervical, dorsal, and lumbar spine (Fig. 2) as well as pain on passive and active mobilization of both hips. The rest of the somatic examination was unremarkable, including no cardiovascular symptoms.

Biologically, the assessment was in favor of an inflammatory profile with a C-reactive protein at 87 mg/l, a fibrinogen level at 7 g/l, hypochromic microcytic anemia at 8 g/dl, thrombocytosis at 500,000 / mm3, electrophoresis of serum proteins found hypoalbuminemia at 30 g/l, hyper alpha 1 and alpha 2 globulins. The HLA B27 was positive. Pro-BNP (brain natriuretic peptide) assay was frankly positive at 885 pg/ml.

**Fig. 3:**
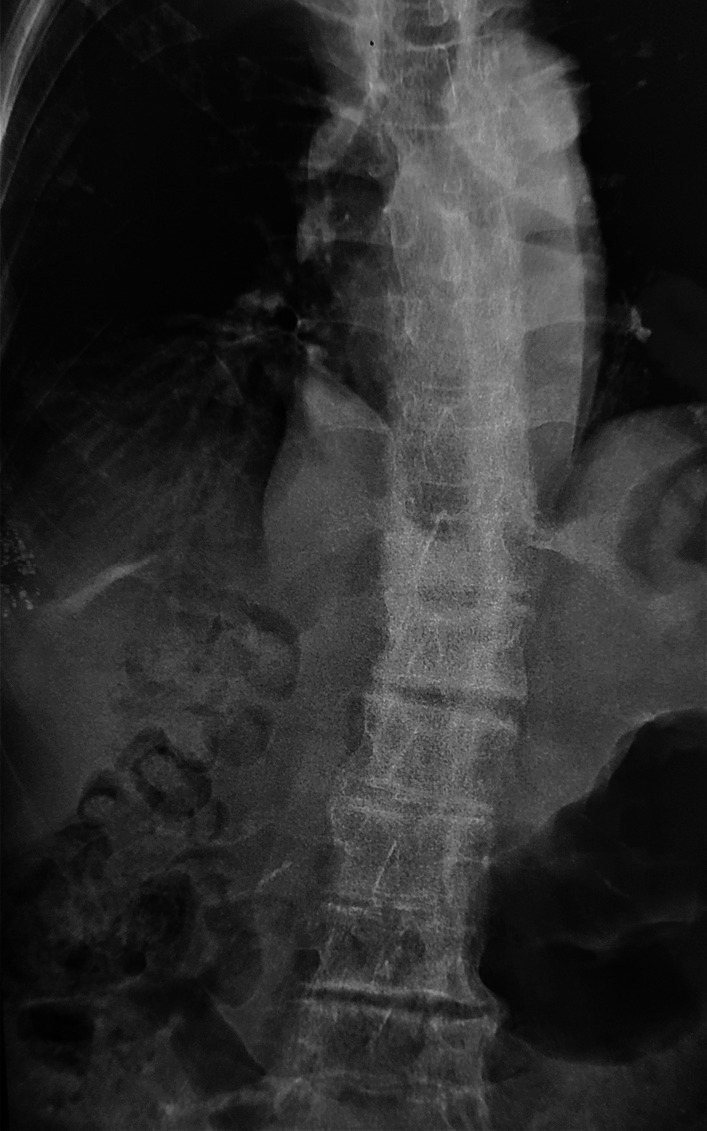
Frontal X-ray of the lumbar spine: syndesmophytes, joint pinching, and diffuse bone demineralization

X-rays were performed (Fig. 3), showing a bamboo spine appearance at the level of the thoraco-lumbar spine, accentuated kyphosis.

**Fig. 4:**
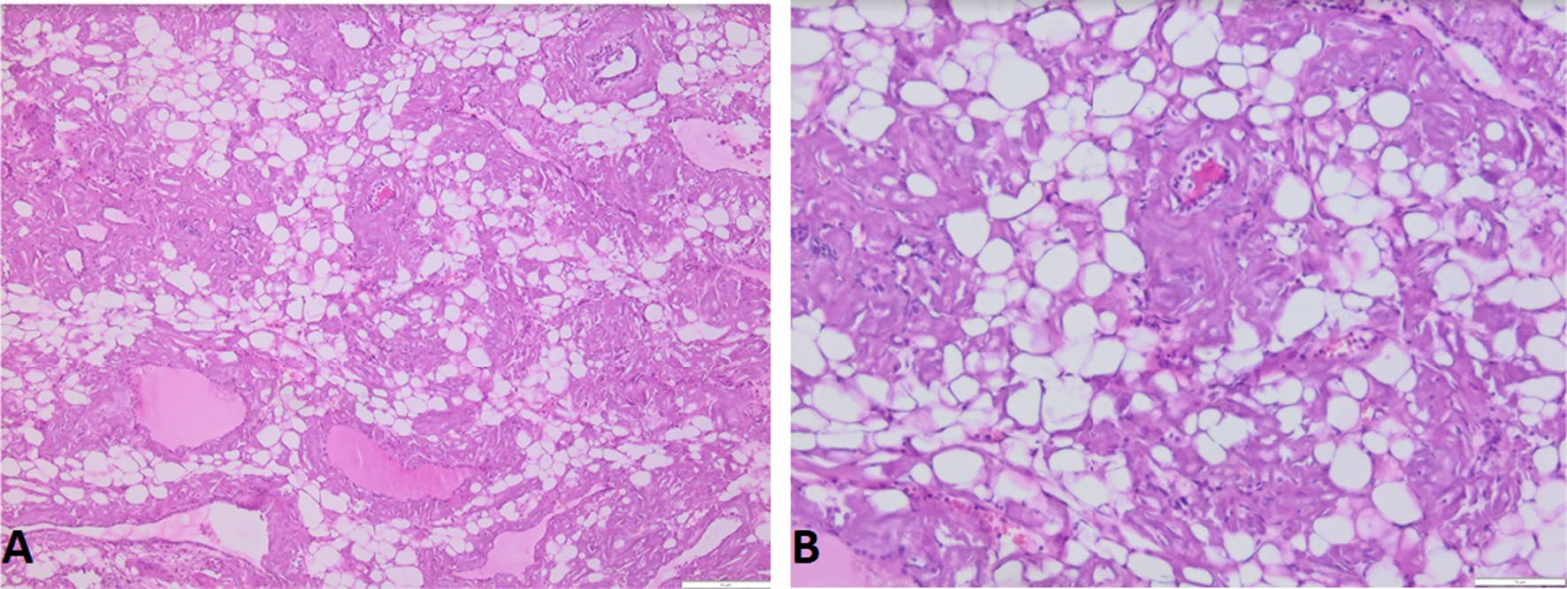
Histological section of a *thyroid parenchyma*: The interstitial tissue is enlarged by an abundant amyloid deposit comprising clusters of mature adipocytes interposed between the thyroid vesicles (**A** H&E staining ×200), (**B** H&E staining ×100)

**Fig. 5:**
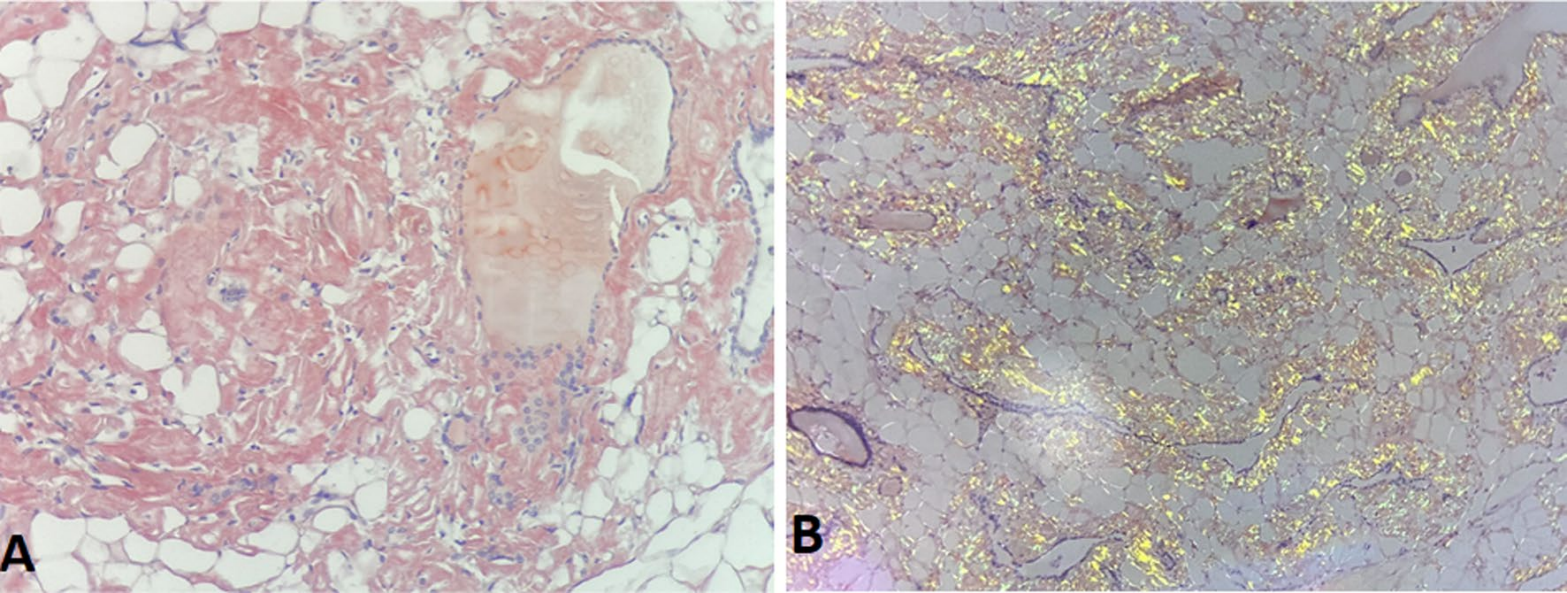
Histological section of a *salivary parenchyma* in special Congo red staining marks the deposition in brick red (**A** Congo red ×200) and characteristic yellow–green birefringence in polarized light (**B** polarized light ×100)

**Fig. 6:**
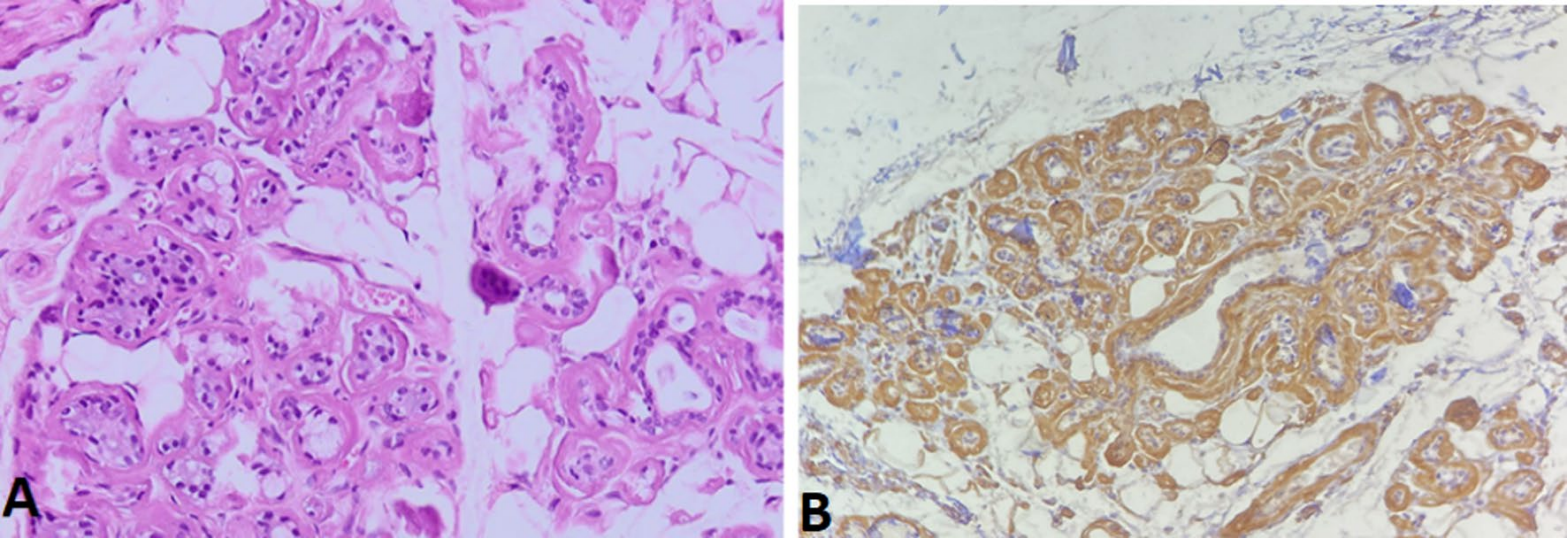
Histological section of a *salivary parenchyma*: extracellular areas of anhistic, amorphous material. Amyloid deposits (**A** H&E staining ×200) with positive staining by anti-SAA antibody in immunohistochemistry (**B** anti-SAA antibody ×100)

Histology of thyroid tissue (Fig. 4) after thyroidectomy found a thyroid parenchyma where the interstitial tissue is enlarged by an abundant amyloid deposit comprising clusters of mature adipocytes interposed between the thyroid vesicles. A biopsy of the accessory salivary glands was performed (Figs. 5 and 6), which revealed amyloid deposits with positive staining by anti-serum amyloid A (SAA) antibody in immunohistochemistry.

**Fig. 7:**
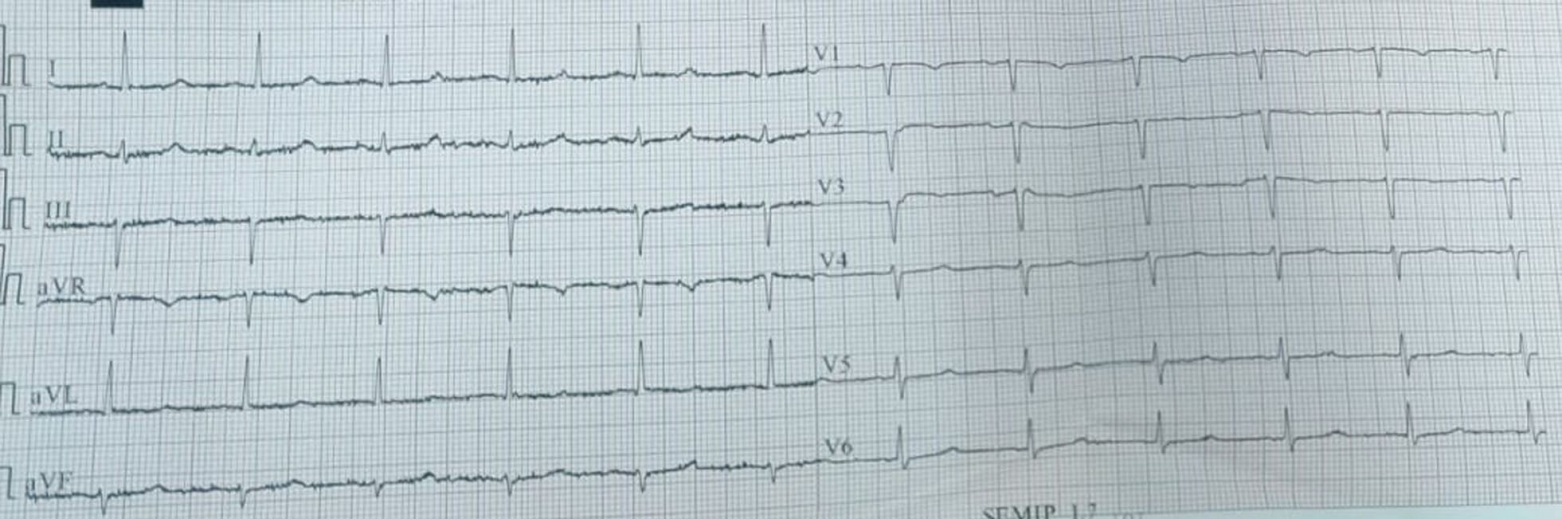
The electrocardiogram

**Fig. 8:**
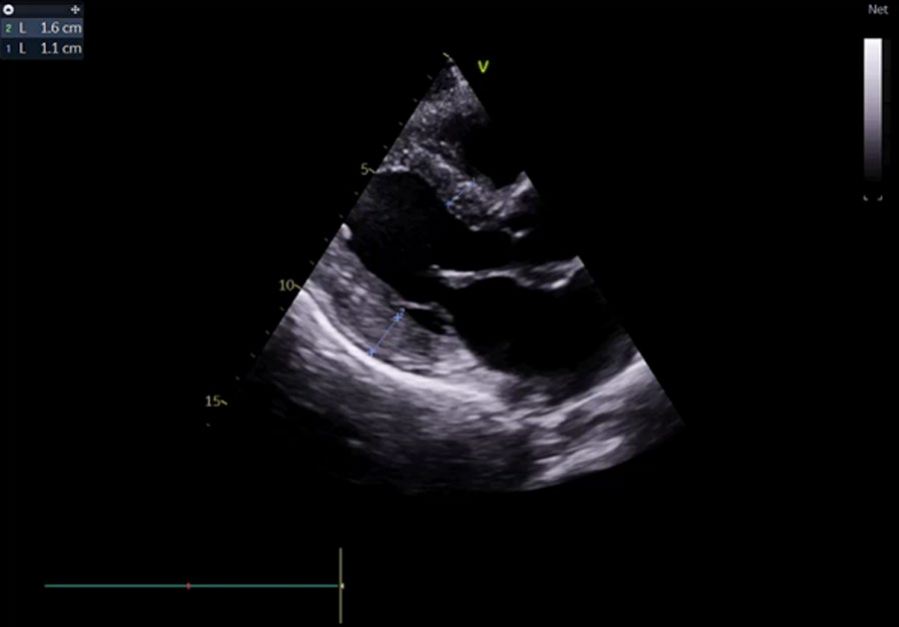
Mean LV hypertrophy

**Fig. 9:**
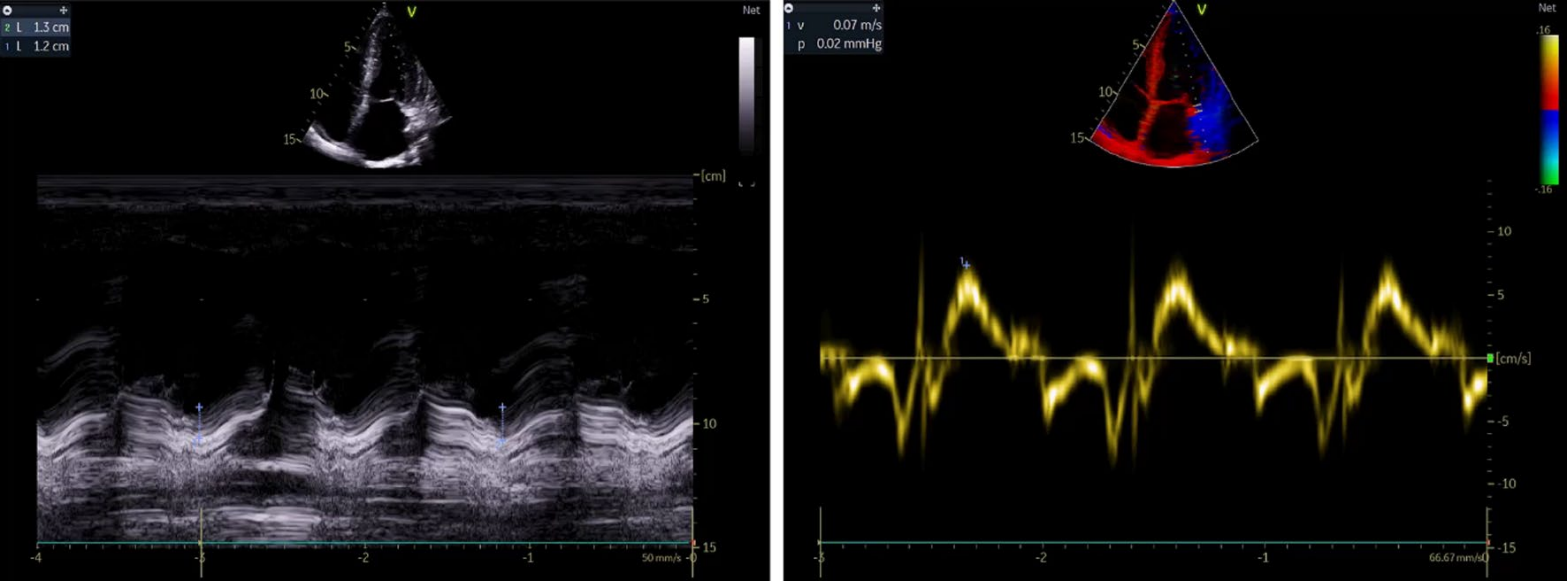
Alteration of LV longitudinal function

**Fig. 10:**
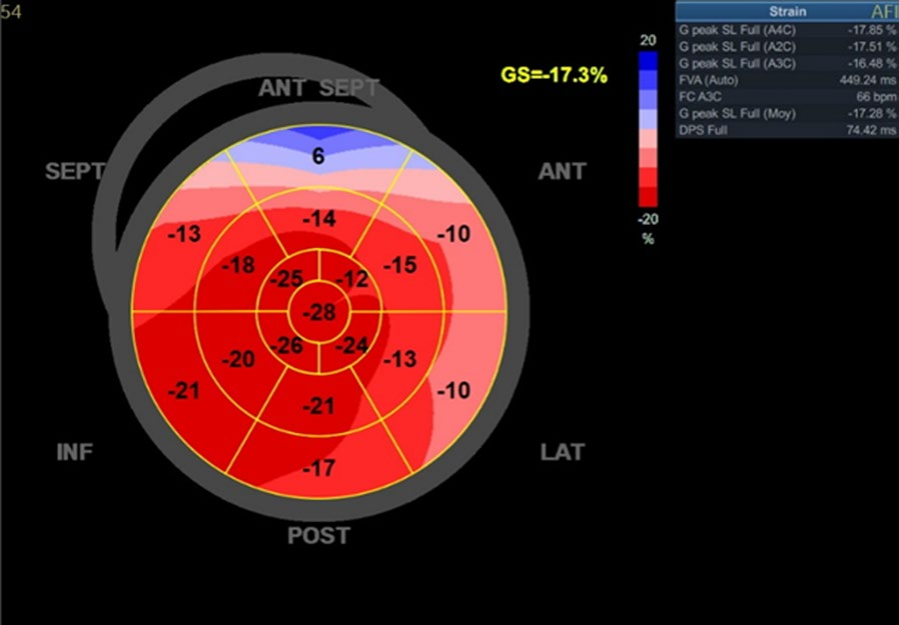
Alteration of the global longitudinal strain of the LV at − 17.3%

**Fig. 11:**
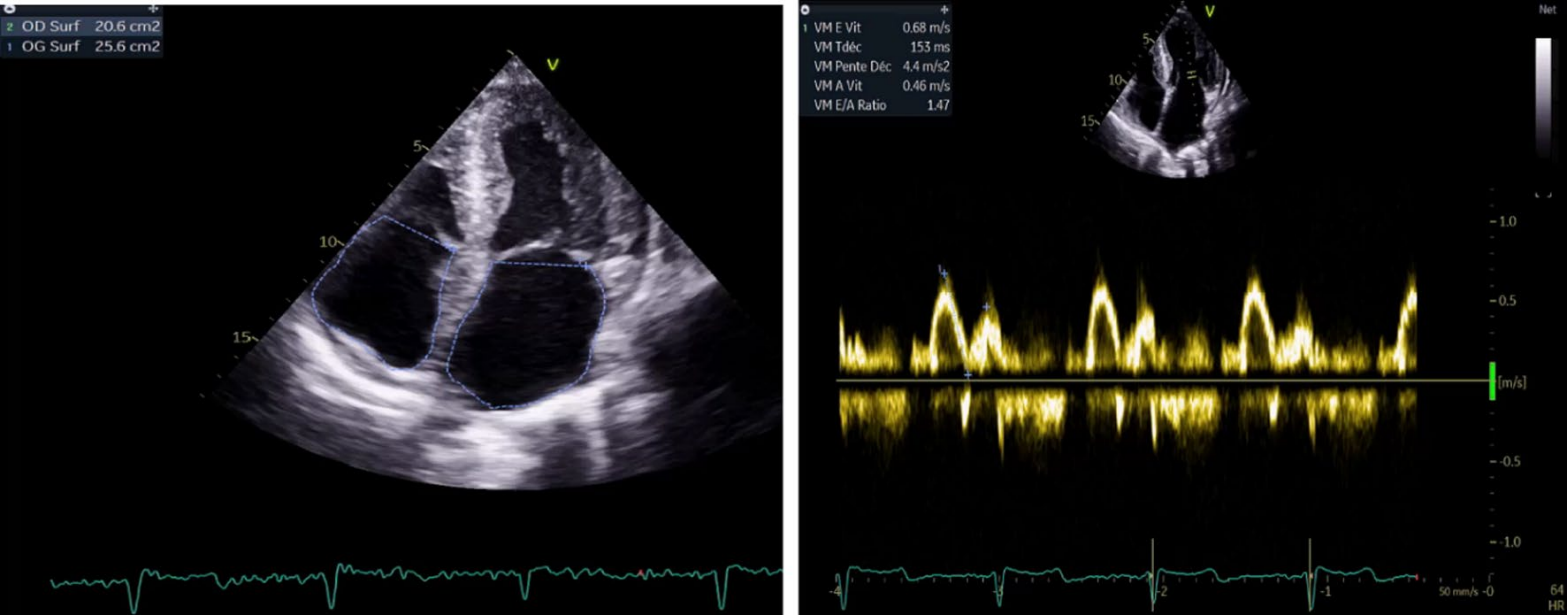
Pseudonormal mitral profile and biatrial dilatation

**Fig. 12:**
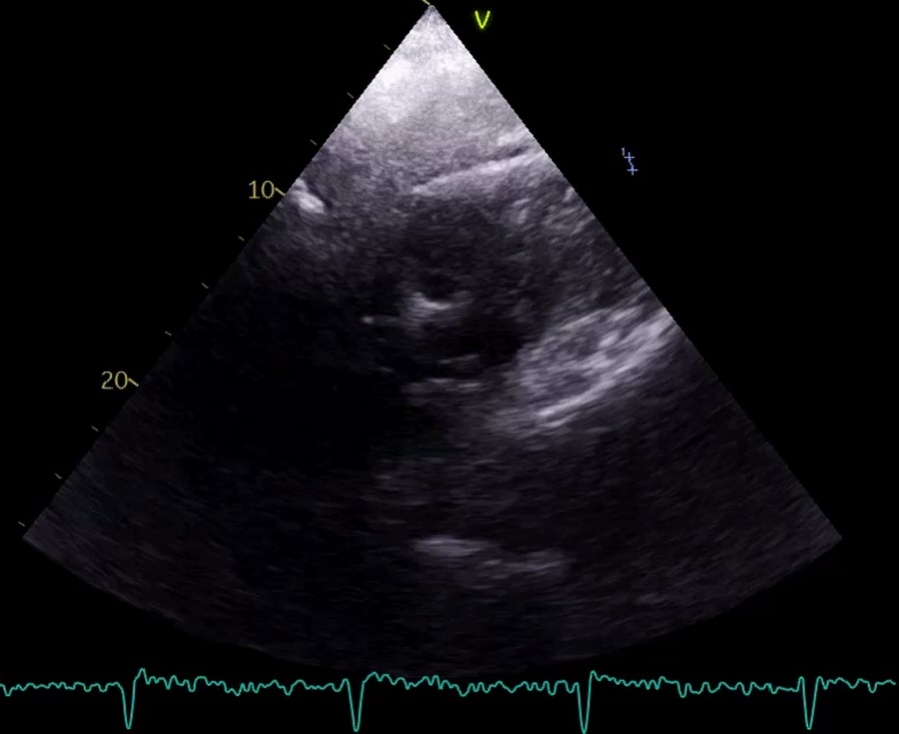
Low abundance pericardial effusion near the right cavities

A cardiac assessment was carried out. The electrocardiogram (ECG) (Fig. 7) revealed a sinus rhythm at 75 bpm, with diffuse microvoltage, pseudo-infarct pattern, and negative T-wave from V1 to V3. On transthoracic echocardiography the left ventricle (LV) was normal sized (indexed left ventricular end-diastolic diameter (ILVEDD) = 40 mm/m^2^), hypertrophied (interventricular septum (IVS) = 11 mm, posterior wall = 16 mm) (Fig. 8), with a normal radial systolic function (left ventricular ejection fraction (LVEF) = 65%), an altered longitudinal function with a mitral annular plane systolic excursion (MAPSE) = 13 mm, and an S’VG = 6 cm/s (Fig. 9), and a global longitudinal systolic strain altered at -17.3% (Fig. 10). We also noted a pseudonormal mitral profile, with biatrial enlargement (left atrium (LA) = 26 cm^2^, right atrium (RA) = 21 cm^2^) (Fig. 11). The right ventricular (RV) was not dilated, not hypertrophied, with a normal systolic function. There was a minimal pericardial effusion next to the right cavities (Fig. 12).

The patient received anti-TNF (tumor necrosis factor) treatment, and after 3 months of treatment, there was a significant biological improvement of the inflammatory syndrome.

## Discussion

Ankylosing spondylitis is a chronic immune-mediated inflammatory arthritis included in the so-called group of spondyloarthritis (SpA). The axial skeleton and the sacroiliac joints are the primary targets of this disease in males in their third decade of life [1]. Other disabling manifestations, such as inflammatory bowel disease, uveitis, and amyloidosis, may occur in addition to skeletal involvement [2].

Inflammatory amyloidosis, also known as AA amyloidosis (amyloid associated), is a form of generalized amyloidosis, just like AL amyloidosis (immunoglobulinic) and hereditary amyloidosis. In AA amyloidosis, the amyloid protein is the AA protein, which is derived by cleavage from the "serum amyloid associated protein" (SAA), one of the major proteins in the reaction inflammatory [3]. The diagnosis of amyloidosis is based on clinical organ involvement and histological evidence of target organ showing deposition of abnormally folded proteins leading to organ dysfunction. Amyloid deposits are formed from globular, soluble proteins, which undergo misfolding and, subsequently, aggregate into insoluble fibrils, or proteins may also have an intrinsic tendency to form amyloid in the absence of misfolding [4]. The spleen and liver are the first places where AA amyloid deposits appear [5]. Nevertheless, despite significant amyloid infiltration, splenic and hepatic involvement remain asymptomatic for a long time or only lead to mild liver function abnormalities. On the other hand, the deposition of AA amyloid fibrils in the mesangium and in the glomerular capillary walls in the kidney invariably leads to proteinuria, nephrotic syndrome, and the progressive development of renal failure, which are therefore the main features of the presentation of AA amyloidosis [6].

Our patient had undocumented nephropathy which was probably due to renal amyloidosis. Amyloid infiltration of the thyroid gland is possible; however, thyroid amyloid deposits are rarely large enough to result in clinically recognizable goiter; less than 150 cases of amyloid goiter have been described in the literature [7]. Amyloid goiters grow rapidly, while most patients present with a euthyroid state like our patient, cases of hypothyroid, and hyperthyroid states have also been reported [8]. Only four cases of amyloid goiter secondary to ankylosing spondylitis have been described in the literature [7, 9–11].

The prevalence of cardiac involvement is unknown in published studies and is likely underestimated [12]; although deposition of AA amyloid can be observed by echocardiography in approximately 10% of patients, restrictive cardiomyopathy progressing to heart failure rarely develops even in stage late in the disease [13]. Diagnosing cardiac involvement in AA amyloidosis can be challenging [14]. Despite an extensive literature search of noninvasive imaging modalities for the diagnosis of cardiac AA amyloidosis, no historical studies or convincing case reports were identified. In one study, clinical heart failure was identified in only one of 374 patients with AA amyloidosis and significant LV hypertrophic on transthoracic echocardiography [15]. In another study of 199 patients with AA amyloidosis, 23 (12%) had heart failure, and cardiac biopsies were performed in 13, all of which showed AA amyloid deposits [16].

The diagnosis of cardiac amyloidosis (CA) is difficult and requires the combination of several clinical data, different imaging, and histological modalities (endomyocardial biopsy) [14]. Patients with CA present with a variable clinical presentation that can range from asymptomatic LV dysfunction detected as part of the assessment of systemic amyloidosis, as is the case of our patient, to refractory heart failure or cardiogenic shock. Usually dyspnea, edema, palpitations, or syncope is the main symptom revealing cardiac involvement [17]. Since the ECG is an easy and accessible examination, it makes it possible to orient toward a diagnosis of cardiac amyloidosis. In a study carried out in 2013 [14], on the various most sensitive and specific electrical aspects in the event of cardiac amyloidosis, we find: low voltage on limb leads, atrial arrhythmia, atrioventricular block, and pseudo-infarct pattern. In cardiac amyloidosis, limb leads with low-voltage and pseudo-infarct pattern were more common.

Echocardiography, which is widely available, has greatly improved the diagnosis of CA after the introduction of strain analysis; despite this, its sensitivity and specificity remain low [14]. In all patients with unexplained LV hypertrophy and clinical suspicion of cardiac amyloidosis, it is recommended to perform full 2D echocardiography, which includes quantitative tissue Doppler and longitudinal strain analysis [18]. Echocardiographic evaluation for signs of amyloidosis such as myocardial wall hypertrophy and biatrial dilation is helpful [17]. The presence of moderate to severe LV thickening (wall thickness ≥ 14 mm) with or without right ventricle (RV) hypertrophy should raise suspicion of CA [14]. Granular appearance scintillation of the myocardial walls can be appreciated [18]. Myocardial contractility can be preserved or even supranormal for a long period. Heart failure occurs at a later stage when left ventricular filling is impaired [14]. Taking the ratio of apical longitudinal strain to basal and median longitudinal strain, a value greater than 1.3 predicts the existence of CA. With the presence of the "cherry-like" apical sparing strain associated with other features such as biventricular hypertrophy, atrial enlargement, a restrictive mitral profile and pericardial effusion. In this context, a more in-depth investigation using other more precise diagnostic modalities such as bone scintigraphy, cardiac magnetic resonance imaging (MRI), should be carried out [19].

Echocardiographic parameters should be combined with electrocardiographic, clinical, biomarker, and other imaging findings to maximize diagnostic accuracy [18]. It should be noted that cardiac biomarkers (troponins and pro-BNP (brain natriuretic peptide)) remain one of the important diagnostic parameters. And the evaluation of these biomarkers is an integral part of the management of patients with cardiac amyloidosis [18].

For this, cardiac MRI remains a powerful examination which makes it possible to differentiate cardiac amyloidosis from hypertrophic cardiomyopathy (HCM) depending on the late gadolinium enhancement (LGE) model.

Despite all the progress, the challenge for effective and rapid diagnosis persists. The only sure diagnosis of cardiac amyloidosis requires an endomyocardial biopsy, which remains the gold standard, as it is virtually 100% accurate. Endomyocardial biopsy is necessary when suspected cardiac amyloidosis is an isolated feature or when the type of cardiac amyloid fibril cannot be identified by other means. In practice, in AA amyloidosis we have other histological evidence. Therefore, the need for endomyocardial biopsy is limited. Cardiac amyloidosis has a poor prognosis, but this differs depending on the type of amyloidosis, stage of heart failure, availability, and response to treatment. The treatment takes several parts: The treatment of heart failure (HF) (beta-blocker, angiotensin-converting enzyme (ACE) inhibitor, or angiotensin II receptor antagonist (ARA II), sodium-glucose cotransporter 2 (SGLT2) inhibitors, or others), which represents the main component, must be used with caution given the risk of complications and harmful drug interactions. The primary goal of treatment for HF is to maintain adequate filling pressures with the balancing peripheral edema and renal failure. Therapies act on the production of amyloid fibril precursor proteins, and new strategies to inhibit the formation of amyloid fibrils or to directly target amyloid deposits. Heart transplantation, although rarely feasible, can be very effective in carefully selected patients. Education, patient involvement, and support are essential for successful management [20]. And finally, the treatment of AA amyloidosis aims to reduce the production of AAS. In addition to etiological treatment with corticosteroids and immunosuppressants, treatment with monoclonal antibodies directed against cytokines, in particular TNF and interleukin-6 (IL-6), is effective in many cases. This type of treatment is targeted not at the level of the amyloid fibrils themselves but rather at the level of the messengers in the acute phase response [21].

## Conclusions

AA amyloidosis is rare in ankylosing spondylitis; it is a very serious complication due in particular to the risk of renal damage. Care should be taken in case of cardiac involvement, exceptional in AA amyloidosis, or other unusual organ involvement in AA amyloidosis, such as involvement of the thyroid gland. Our case shows that even when faced with an asymptomatic patient, cardiac involvement must be detected, especially when amyloidosis affects several organs.

## Data Availability

Not applicable.

## Abbreviations

AS: Ankylosing spondylitis
AA: Secondary amyloidosis
RA: Rheumatoid arthritis
CA: Cardiac amyloidosis
BNP: Brain natriuretic peptide
SAA: Anti-serum amyloid A
ECG: Electrocardiogram
LV: Left ventricle
ILVEDD: Indexed left ventricular end-diastolic diameter
IVS: Interventricular septum
LVEF: Left ventricular ejection fraction
MAPSE: Mitral annular plane systolic excursion
LA: Left atrium
RA: Right atrium
RV: Right ventricular
TNF: Tumor Necrosis Factor
MRI: Magnetic resonance imaging
HCM: Hypertrophic cardiomyopathy
LGE: Late gadolinium enhancement
HF: Heart failure
ACE: Angiotensin-converting enzyme
ARA II: Angiotensin II receptor antagonist
SGLT2: Sodium-glucose cotransporter 2
IL-6: Interleukin-6
